# Evaluation of the Value of Perfusion-Weighted Magnetic Resonance Imaging in the Differential Diagnosis of Sellar and Parasellar Tumors

**DOI:** 10.3390/jcm12082957

**Published:** 2023-04-19

**Authors:** Adrian Korbecki, Weronika Machaj, Justyna Korbecka, Michał Sobański, Maciej Kaczorowski, Paweł Tabakow, Agnieszka Hałoń, Grzegorz Trybek, Przemysław Podgórski, Joanna Bladowska

**Affiliations:** 1 Department of General Radiology, Interventional Radiology and Neuroradiology, Wroclaw Medical University, Borowska 213, 50-556 Wroclaw, Poland; michalsobanski11@wp.pl (M.S.); przemyslaw.podgorski@umw.edu.pl (P.P.); joanna.bladowska@umw.edu.pl (J.B.); 2 Department of Physiology and Pathophysiology, Wroclaw Medical University, Chalubinskiego 10, 50-368 Wroclaw, Poland; wnzmachaj@gmail.com; 3 Department of Neurology, Wroclaw Medical University, Borowska 213, 50-556 Wroclaw, Poland; j.e.korbecka@gmail.com; 4 Department of Clinical and Experimental Pathology, Wroclaw Medical University, Marcinkowsiego 1, 50-368 Wroclaw, Poland; maciej.kaczorowski@umw.edu.pl (M.K.); agnieszka.halon@umw.edu.pl (A.H.); 5 Department of Neurosurgery, Wroclaw Medical University, Borowska 213, 50-556 Wroclaw, Poland; p.tabakov@wp.pl; 6 4th Military Clinical Hospital in Wroclaw, Rudolfa Weigla 5, 50-981 Wroclaw, Poland; g.trybek@gmail.com; 7 Department of Oral Surgery, Pomeranian Medical University in Szczecin, 70-111 Szczecin, Poland

**Keywords:** magnetic resonance imaging, perfusion-weighted imaging, sellar tumors, pituitary adenomas

## Abstract

The purpose of this study was to assess the value of perfusion-weighted imaging (PWI) in the differential diagnosis of sellar and parasellar tumors, as an additional sequence in the magnetic resonance imaging (MRI) protocol. Analysis was based on a substantial group of subjects and included 124 brain and pituitary MRI examinations with a dynamic susceptibility contrast (DSC) PWI sequence. The following perfusion parameters were determined for the tumors: relative cerebral blood volume (rCBV), relative peak height (rPH) and relative percentage of signal intensity recovery (rPSR). To ensure greater repeatability, each of the aforementioned parameters was calculated as: arithmetic mean of the values of the whole tumor, arithmetic mean of the maximum values on each axial slice within the tumor and maximum values derived from the whole tumor. In our study, we established that meningiomas compared to both non-functional and hormone-secreting pituitary adenomas (pituitary neuroendocrine tumors—PitNET) had significantly higher values of rCBV with cut-off points set at 3.45 and 3.54, respectively (mean rCBV). Additionally, meningiomas presented significantly higher maximum and mean maximum rPH values compared to adenomas. DSC PWI imaging adds significant value to conventional MRI examinations and can be helpful in differentiating equivocal pituitary tumors.

## 1. Introduction

**Figure 1:**
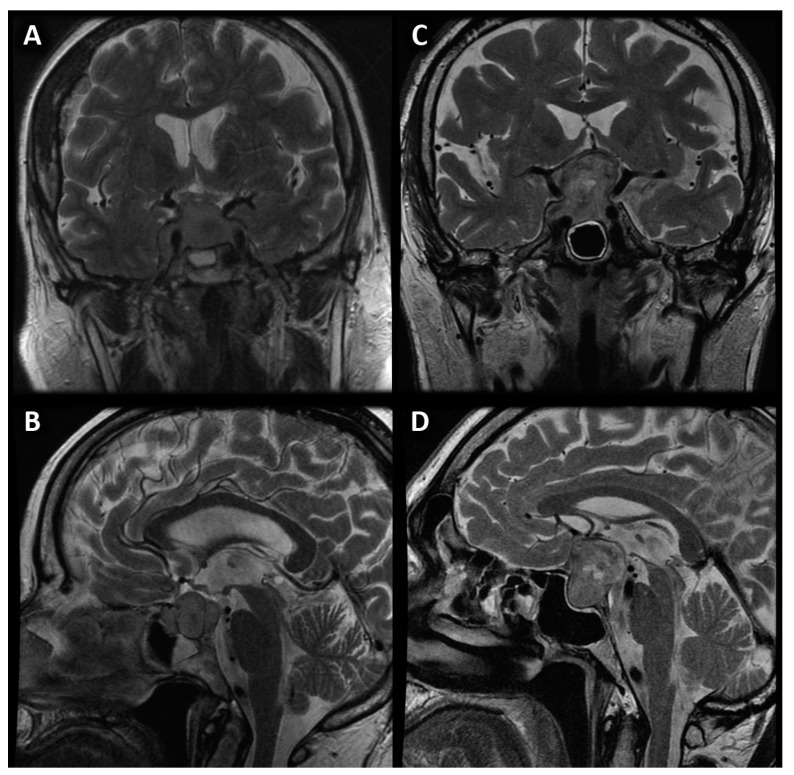
Coronal (**A**) and sagittal (**B**) image of sellar meningioma compared with coronal (**C**) and sagittal (**D**) image of pituitary macroadenoma with suprasellar extension and cystic parts.

Tumors of the sellar and parasellar region represent a heterogeneous group of intracranial primary neoplasms and constitute approximately 10%–15% of central nervous system (CNS) tumors [1,2]. The most common are adenomas (which are interchangeably referred to as pituitary neuroendocrine tumors—PitNETs), meningiomas and craniopharyngiomas. However, in the sellar region may also occur other, less frequent lesions, such as Rathke’s cleft cysts, gliomas, metastases or abscesses. Clinical symptoms are often related to secretory malfunction of the pituitary gland and mass effect of the tumor on the adjacent anatomical structures. A proper diagnosis enables appropriate and effective therapy. Currently, the method of choice in the diagnostic process of the sellar and parasellar regions is magnetic resonance imaging (MRI) [3,4]. Conventional MRI sequences (T1-weighted and T2-weighted images, as well as contrast-enhanced T1-weighted sequences) do not always allow unambiguous identification of the type of lesion (Figure 1). Advanced MR techniques such as diffusion-weighted imaging (DWI), perfusion-weighted imaging (PWI) and magnetic resonance spectroscopy (MRS) allow us to obtain additional information, thus improving the accuracy of diagnosis. These techniques are still rarely used in routine clinical practice. The number of publications in the current literature is limited; the results are often contradictory and based on an inadequate number of subjects [5,6,7,8,9,10,11,12,13]. Therefore, the purpose of our study was to assess the value of PWI in the differential diagnosis of sellar and parasellar tumors, as an additional sequence in the MRI protocol in a substantial group of subjects.

## 2. Materials and Methods

### 2.1. Materials

This retrospective study analyzed a total number of 124 brain and pituitary gland MRI examinations, with the DSC PWI magnetic resonance perfusion sequence in their protocol, performed at the Department of General and Interventional Radiology and Neuroradiology at the University Clinical Hospital in Wroclaw from 2008 to 2019. Women represented 46.8% (58) of the group, while men represented 53.2% (66). The mean age and standard deviation (SD) was 57 ± 18 years. Histopathological examination confirmed the diagnosis of 115 out of 124 tumors (92.7%). The remaining nine lesions (7.3%) were assessed twice by an experienced neuroradiologist with more than 20 years’ clinical practice. The 124 examinations were divided into 15 groups (Table 1).

Exclusion criteria were as follows: tumors with a size of less than 1.0 cm in the anterior-posterior and cranio-caudal direction, severe movement artifacts and artifacts from calcifications and hemorrhage within the tumor, which interfered with perfusion parameters values. We also avoided taking measurements in the vicinity of the internal carotid artery or middle cerebral artery to prevent disturbances related to contrast flow in the vessels.

This study was performed under the guidance of and with the approval of the local University Ethics Committee for conducting research involving humans. Each patient signed a consent form to participate in the study.

### 2.2. Methods

The MR examinations were performed on a GE Signa Hdx 1.5T scanner with a 16-channel coil dedicated to the head, neck and spine (HNS—head, neck, spine). Standard sequences for pituitary MR examination included T1-, T2-, and T1-weighted after intravenous administration of gadolinium contrast. Standard magnetic resonance imaging sequences were used to assess the dimensions (AP, TR, CC) of the lesion, morphology (solid, fluid) and the presence foci of the hemorrhage.

An additional protocol included in the study was DSC-PWI. This technique is based on changes in the magnetic field after gadolinium contrast administration in T2* images. The precise parameters of the sequence are as follows: TR = 1900 ms, TE = 80 ms, FOV = 30 cm, flip angle = 90 degrees, matrix size = 192 × 129, slice thickness 8 mm with no gap, gadolinium contrast dose 0.2 mmol/kg body weight, flow rate 5 mL/s (administered through the antecubical vein). The first 10 seconds of the scan were acquired before contrast administration in order to obtain the baseline of DSC-PWI examination.

### 2.3. Perfusion Measurements 

**Figure 2:**
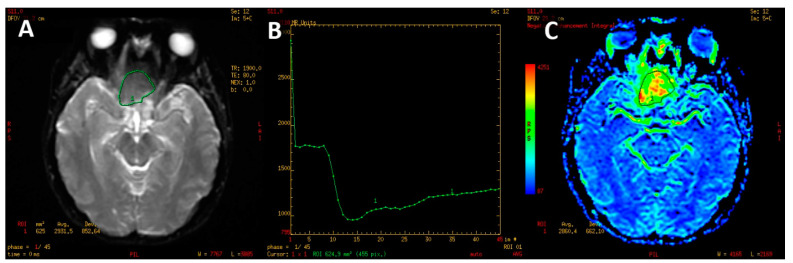
PWI image of macroadenoma (**A**). T2* signal intensity curve (**B**) with marked decline of signal intensity during the first pass on contrast agent with slow, partial return to the baseline. Color-coded map of cerebral blood volume (**C**). High CBV is presented as yellow and red colors.

The PWI sequence was post-processed on a dedicated GE Medical Systems ADW 4.6 diagnostic workstation with Funcool software provided by the manufacturer. For each study, a color-coded perfusion map and perfusion curve were calculated (Figure 2).

#### 2.3.1. Measurements of Normal-Appearing White Matter

**Figure 3:**
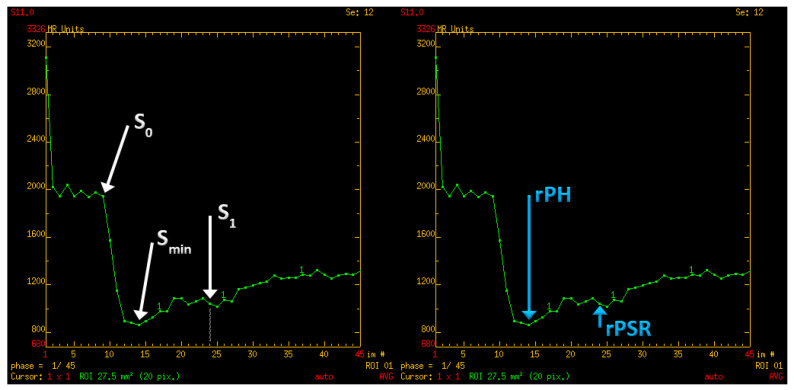
Perfusion curve parameters: S_0_—A baseline signal intesity; S_min_—minimal signal values during first pass of gadolinium contrast; S_1_—signal intensity recovery 14 s after the beginning of the signal drop; rPH—relative peak height; rPSR—relative percentage of signal recovery.

In the first step of the study, perfusion parameters were determined for normal-appearing white matter. A region of interest (ROI) was set in the right frontal lobe (a round area of approximately 500 mm^2^). The parameters measured were as follows: CBV (cerebral blood volume), S_0_ (signal intensity of the baseline of the perfusion curve), S_min_ (minimal signal value during the first pass of gadolinium contrast), S_1_ (signal intensity recovery 14 s after the beginning of the signal drop—the first passage of contrast through the vascular bed takes approximately 10 to 14 s). Subsequently, PH (peak height) and PSR (percentage of signal intensity recovery) were calculated with the following equations: PH = S_0_ − S_min_; PSR = (S_1_ − S_min_)/(S_0_ − S_min_) (Figure 3). These perfusion measurements were necessary as reference points to obtain normalized (relative) values afterward.

#### 2.3.2. Measurements of Tumors of the Sellar and Parasellar Regions

**Figure 4:**
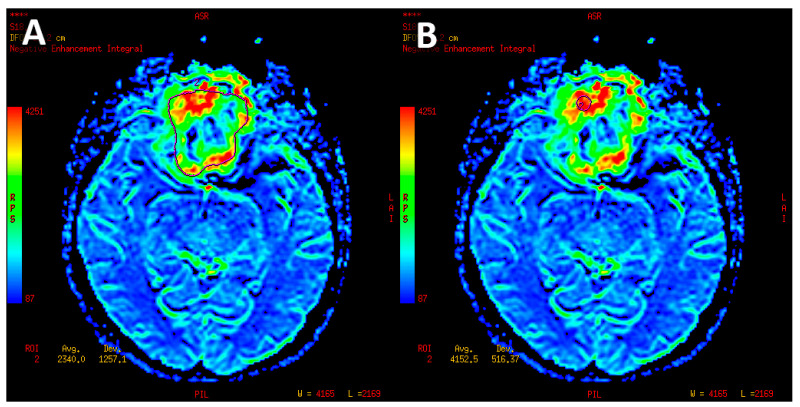
Meningioma with manually set region of interest outlining the tumor margins on axial slice used to measure mean CBV (**A**). Circular ROI about 58 mm^2^ inserted in the hyperperfused part of lesion used to measure maximum CBV (**B**).

In the next step of the study, sellar and parasellar lesions were evaluated. The whole tumor was manually outlined on each axial section as an ROI. Regions with calcifications and hemorrhages or adjacent vessels were excluded. On each slice, the perfusion parameters were measured. Subsequently, these measurements were divided by the parameters received for-normal appearing white matter to obtain relative values (rCBV, rPH, rPSR). To ensure greater repeatability, perfusion values for each tumor were determined with three different methods as follows:

- Mean perfusion parameters for the whole tumor (labeled as rCBV’1)—the arithmetic mean of the perfusion values collected by outlining the tumor with ROIs on each axial slice (Figure 4A).
- Mean of maximum perfusion parameters (labeled as rCBV’2)—the arithmetic mean of the maximum perfusion values collected by outlining the regions with the highest values with circular ROIs (about 30–60 mm^2^) on each axial slice of the tumor (Figure 4B).
- Maximum perfusion values (labeled as rCBV’3)—the maximum values collected from the whole tumor with a circular ROI (about 30 mm^2^–60 mm^2^) (Figure 4B).

### 2.4. Statistical Analysis

Statistical analysis was performed in the R statistical platform (version 4.1.0). Initially, analysis was conducted to identify pairs of tumors with statistically significant differences.

Box-type charts were created to preface the distribution of individual variables for the analyzed tumors. At that point, the receiver operating characteristic (ROC) curves for tumor pairs (considering tumors with a number of observations > 10) were used to verify the usefulness of the magnetic resonance perfusion parameters in the differential diagnosis of tumors. Optimal cut-off points were determined, as well as a number of indices determining the convenience of diagnostic tests (area under the curve—AUC, positive predictive value, negative predictive value, accuracy, specificity, sensitivity). 

The analyzed parameters were: rCBV (all measurement variants), rPH (all measurement variants), rPSR (all measurement variants), age, TR, CC and AP. Tumor groups consisted of <5 subjects that were not analyzed for statistical significance due to insufficient abundance; for these, we determined only the arithmetic mean of the measurements.

Statistical inference was based on a significance level of *p* = 0.05 throughout the analysis.

## 3. Results 

**Figure 5:**
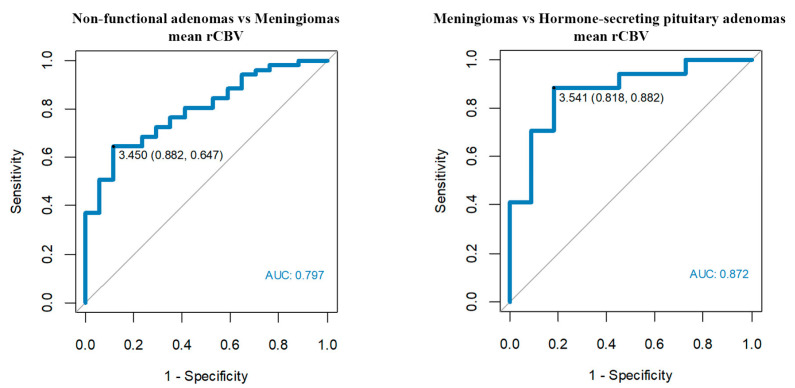
Optimum rCBV cut-off points among meningiomas, non-functional pituitary adenomas and hormone-secreting adenomas.

In our study, non-functional pituitary adenomas (*n* = 51) showed mean rCBV = 2.87 for the whole tumor (range 0.29–6.95), maximum rCBV = 4.73 (range 0.43–20.38). Similarly, adenomas with endocrine function (n = 11) showed mean rCBV = 2.61 (range 1.09–4.91) and maximum rCBV = 4.33 (range 1.34–10.54). In comparison, meningiomas received higher values, both mean and maximum rCBV: 5.09 (range 2.11–11.33) and 8.25 (range 2.99–25.17), respectively. Differences in rCBV values were statistically significant between meningiomas and all groups of adenomas. We established the subsequent cut-off points with the highest accuracy (Table 2, Figure 5):

- Mean rCBV’1 > 3.45 enables us to differentiate meningiomas from non-functional pituitary adenomas with a sensitivity of 65% and a specificity of 88%.
- Mean rCBV’1 > 3.54 enables us to differentiate meningiomas from hormone-secreting adenomas with a sensitivity of 88% and a specificity of 82%.

In the case of the rPH perfusion parameter, non-functional pituitary adenomas showed mean rPH = 1.63 (range 0.63–5.92) for the whole tumor and maximum rPH = 2.51 (range 0.28–8.61). Adenomas with endocrine function received similar values of mean rPH = 1.51 for the whole tumor (range 0.2–3.3) and maximum rPH = 2.32 (range 0.2–4.35). Consistently, rPH values of meningiomas were higher than PitNETs, with mean rPH = 2.28 (range 0.99–5.60) and maximum rPH = 3.90 (range 1.07–9.01). According to ROC curves, the maximum rPH and mean of maximum rPH values of meningiomas were significantly higher when compared to non-functional adenomas and hormone-secreting adenomas. 

**Figure 6:**
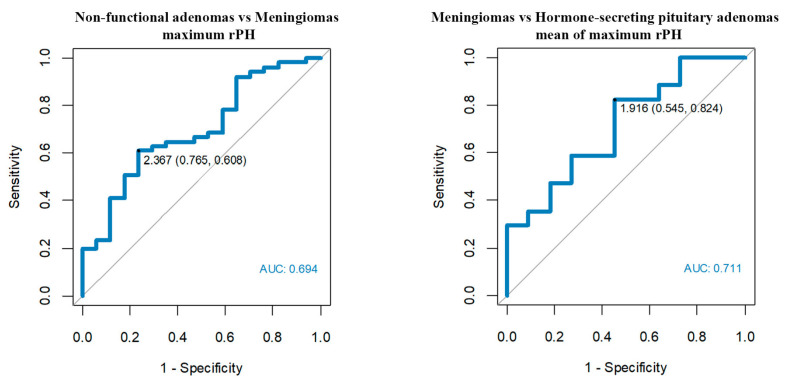
Optimum rPH cut-off points among meningiomas, non-functional pituitary adenomas and hormone-secreting adenomas.

The most accurate cut-off points were (Table 3, Figure 6):

- Maximum rPH’3 > 2.37 enables us to differentiate meningiomas from non-functional adenomas with a sensitivity of 61% and a specificity of 76%.
- Mean of maximum rPH’2 > 1.92 enables us to differentiate meningiomas from hormone-secreting adenomas with a sensitivity of 82% and a specificity of 55%.

In our study, we also evaluated the values of perfusion parameters of both invasive and non-invasive PitNET. Nevertheless, we found no correlations between perfusion parameter values and the invasive character of the tumor. 

Furthermore, we determined that larger dimensions of meningiomas (AP, TR and CC) were associated with higher values of rPH. The mean values of the size of the tumors are presented in table number 4 (Table 4). No other correlations were found between tumor size and perfusion parameters.

**Figure 7:**
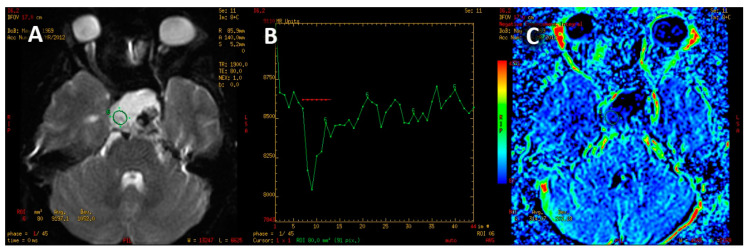
Adamantinomatous craniopharyngioma. PWI image (**A**). T2* signal intensity curve with rapid return, exceeding the baseline signal (**B**). Color-coded map of cerebral blood volume with minor perfusion values within the tumor (**C**).

Measurements of perfusion parameters for the adamantinomatous type of craniopharyngiomas (n = 9) were as follows: mean rCBV = 1.57; max rCBV = 2.63; mean rPH = 0.71, max rPH = 1.7 (Figure 7). These rCBV values were significantly lower compared to meningiomas (*p* = 0001–0.005).

On the other hand, the papillary type of craniopharyngiomas (n = 4) revealed the following values of perfusion parameters: mean rCBV = 2.25; max rCBV = 5.24; mean rPH = 1.94, max rPH = 5.06. Values are shown in Table 3 with the results of the other tumors group consisting of ≤ 5 subjects (Table 5).

**Figure 8:**
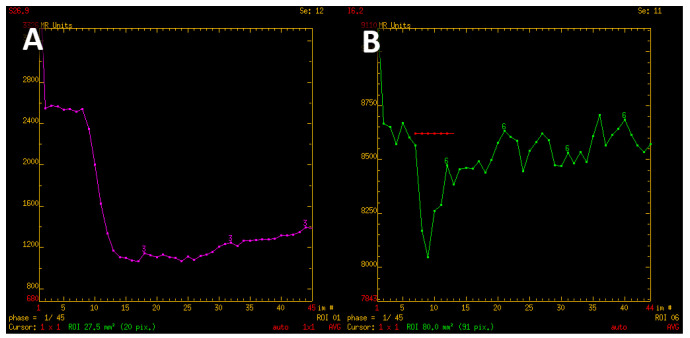
T2* signal intensity curve characteristic for meningioma, with minimal return to the baseline (**A**). Contrary, signal intensity curve of adamantinomatous craniopharyngioma shows rapid return and even exceeds the baseline values (**B**).

It is important to emphasize that rPSR was the perfusion parameter in which no statistically significant correlations were found, despite the fact that in qualitative assessment there were visible differences in the profile of the perfusion curve. For instance, the adamantinomatous type of craniopharyngiomas and lymphomas showed a rapid return of signal and often exceeded the baseline; meningiomas and PitNETs showed a high drop of the signal with a slow or no return of signal to the baseline (Figure 8). For all of the measured variants of rPSR parameters, the *p* value was > 0.05, in particular, for the mean perfusion parameters (*p* = 0.285), mean of maximum perfusion parameters (*p* = 0.154) and maximum perfusion values (*p* = 0.150).

There was only one statistically significant correlation related with age. Patients over 54 years old more often have pituitary adenomas without endocrine function than secretory pituitary PitNETs (sensitivity 86%, specificity 45%).

## 4. Discussion

The main objective of the study was to assess the value of PWI as an additional sequence in the diagnostic protocol of sellar and parasellar tumors. PWI is a technique that enables the imaging and assessment of cerebral flow hemodynamics at the capillary level. The most frequently used imaging method is DSC MRI, which exploits the fluctuations in magnetic field in T2- or T2*-weighted images, during the first passage of a paramagnetic contrast agent through the vascular bed. Results are presented with a color perfusion map and a perfusion curve graph presenting T2* signal values after the first pass of contrast media. The perfusion method allows us to assess parameters such as rCBV, rPH and rPSR. In this study, each parameter was calculated as the arithmetic mean of perfusion values from all ROIs (outlined on each axial slice), the arithmetic mean of the maximum values from circular ROIs (about 30 mm^2^–60 mm^2^, on each axial slice) and the maximum values in the tumor. Our intent was to increase the accuracy of the obtained results.

The methods of measuring perfusion parameters varied in the literature. In this study and also in research conducted by Bladowska et al., mean rCBV, rPH and rPSR values were obtained by contouring the whole tumor on each axial section, while maximum values were measured using several round ROIs with fixed sizes [14]. On the other hand, Zhang et al. and Hakyemez et al. measured the maximum values within the tumor [15,16]. Floriano et al. calculated maximal values by setting a ROI in the peripheral parts of the tumor [17]. Zengyi Ma et al. performed measurements on at least three cross-sections in the central part of the tumor (in which the tumors had the largest diameter) [18]. The size of the ROI in the above studies also varied from 3 mm^2^ to 60 mm^2^ [14,15,16,17,18]. It should be stressed that currently there are no widely accepted, definitive guidelines regarding the method of measurement and determining the ROI. Thus, to increase the accuracy of our study and to obtain the most objective, repeatable results, we decided to perform measurements in three different ways, excluding calcifications, hemorrhages and adjacent vessels. The semiautomatic methods of tumor segmentation and calculating perfusion parameters may be more user-independent. However, we intentionally decided to select the ROI manually, as it has great clinical utility, it is quick, it may be performed regardless of machine vendor, and it does not require using third-party, additionally paid software.

The percentage of signal return (PSR) was determined using the equation (S_1_ − S_min_)/(S_0_ − S_min_) that is available in all publications. S_0_ is the baseline signal of the perfusion curve and S_min_ is the lowest signal value during the first pass of contrast. However, there are no explicit recommendations for determining the S_1_ point. Bladowska et al. determined S_1_ as the value of the signal 24 seconds after the start of the perfusion curve [14]. In the Mangla et al. publication, S_1_ is the signal value at the end of the perfusion curve, while in the publication by Neska et al., S_1_ is the value at 24 seconds after S_min_ [19,20]. According to the publications by Cha et al., Mangla et al. and Lupo et al., S_1_ should be the signal value immediately after the first passage of contrast through the vascular bed [19,21,22]. The first decline of the signal corresponds to the first pass of contrast media, and takes approximately 10 to 14 seconds. Moreover, the shape of the perfusion curve of tumors does not always allow us to unequivocally evaluate the end of the first pass of the contrast media. This is why in our study we determined fixed S_1_ point as the signal value 14 seconds after the beginning of the signal slippage—it resembles a safe and reliable solution, convergent with Cha et. al.

The PWI sequence has been widely used in the differential diagnosis of gliomas, lymphomas and metastases, as well as in the assessment of ischemic stroke. It is valuable in differentiating necrotic lesions after radiotherapy from a recurrent tumor [23,24,25,26]. In addition, PWI is convenient for the differentiation between atypical and malignant meningiomas [15,27,28]. It is worth mentioning that the rCBV parameter revealed a positive correlation with the vascular endothelial growth factor (VEGF), which corresponds to neoangiogenesis of gliomas [29].

However, there are only a few reports in the available literature on the usefulness of PWI in differentiating lesions of the sellar and parasellar regions [14]. To our knowledge, this is the first study evaluating the perfusion parameters of pituitary and parasellar tumors on such a substantial and diverse study group, as well as the first to describe certain groups of pathology.

PitNETs are the most common type of pituitary tumors. They account for 10%–15% of primary intracranial tumors [30,31]. In our study, this was also the most numerous group (total n = 74). The perfusion parameters (rCBV, rPH) of PitNETs in our research were lower when compared with the study of Bladowska et al. (mean rCBV = 3.32, maximum rCBV = 5.18, mean rPH = 2.84, maximum rPH = 4.06) [14]. The measurement method of rCBV and rPH value was similar; thus, the difference may be due to the larger group of subjects in our study. 

In our study, meningiomas (n = 17) revealed the highest number of statistically significant correlations. They account for about 36% of all intracranial tumors and 50% of benign primary tumors of the CNS [32]. Meningiomas are hyperperfused tumors without a blood–brain barrier, which is associated with a persistent leakage. As a result, perfusion curves show a significant signal drop and a minimal or no return to the baseline [14,16,33]. The flow of contrast agent during the first passage through the vascular bed is considerable; therefore, meningiomas acquire high values of rCBV and rPH parameters in PWI. 

In some studies, to minimize leakage due to lack of a blood–brain barrier, PWI was performed using a preload bolus of contrast (preload leakage correction method) or correction during postprocessing [20,34]. We did not use such a method according to our experience with the PWI technique, as some tumors may show low perfusion in a study without preload, which is a very useful sign in the differential diagnosis. For example, lymphomas revealed a low rCBV value without preload, and thus lymphomas can be easily differentiated from gliomas [20]. On the other hand, when perfusion examination is performed with a preload, lymphomas show a high value of rCBV, which is also typical of gliomas [20].

The rCBV values of meningiomas have been measured in several available publications [14,15,16,33,35], but only in one were they located in the sellar and parasellar region [14]. The results obtained in our own study are consistent with the results obtained in the aforementioned publications.

PitNETs and meningiomas may present similar appearance on conventional MRI; therefore, differential diagnosis may be challenging. In our study, we established that the rCBV parameter can differentiate both non-functional and hormone-secreting pituitary adenomas from meningiomas (optimal cut-off values rCBV’1 = 3.45 and rCBV’1 = 3.54, respectively) with high sensitivity and specificity (65% and 88% for the first cut-off point; 88% and 82% for the second cut-off point). We recommend these values for clinical practice.

In our research, all rPSR measurements performed for pituitary PitNETs, meningiomas, adamantinomatous type of craniopharyngiomas, metastases and Rathke’s cleft cysts showed no statistically significant correlations. On the contrary, Mangala et al. and Neska et al. demonstrated the clinical utility of the rPSR parameter in differentiating intracranial lymphomas, GBM and metastases [19,34,36]. This may be due to different tumors being assessed or a different S_1_ point on the perfusion curve. Therefore, further studies are needed to assess the suitability of the rPSR parameter and standardization of this technique. In addition, the shape of the perfusion curve varies depending on the tumor type and is indirectly connected to rPSR value. rPSR is dependent on the value of the signal at a particular point in time and does not represent the total shape of perfusion curve. The adamantinomatous type of craniopharyngiomas and lymphomas presented a rapid return of signal and often crossed the baseline, while meningiomas had a significant decline and a minimal or no return of signal to the baseline [14,20].

The rCBV values of Rathke’s cleft cysts were inappropriately high in comparison with the cystic structure of the tumor. In our opinion, this is due to the inclusion of adjacent cavernous sinuses during the measurements, as a result of the minor size of the lesions (mean TR = 1.22 cm; CC = 1.3 cm; AP = 0.86 cm). Therefore, assessment of perfusion parameters of small tumors should be carried out with great caution. In our experience, the perfusion evaluation of a small sellar lesion is inconclusive and may be confusing.

Some groups in our study were represented with an insufficient number of cases to perform a proper statistical analysis. Nevertheless, our additional observations are worth mentioning.

Values of rCBV and rPH of the papillary type of craniopharyngiomas were noticeably higher compared to the adamantinomatous type of craniopharyngiomas (regarding the enhancing solid part of the tumor), which may be useful for differential diagnosis. In addition, the aforementioned values are coincident with the publication of Larkin et al., where the papillary type of craniopharyngiomas was described as a solid, rarely calcified mass, while the adamantinomatous types are described as firm and often calcified tumors [37].

Metastases are a heterogeneous group, which can be divided into hyperperfused and hypoperfused lesions. Although we analyzed single cases of metastases in our study, the values we obtained are consistent with the results found in other publications—metastases from prostate, lung, and renal cancer typically reveal intermediate to high rCBV values [23,35,36,38,39]. 

Gliomas and lymphomas were also single tumors in our study, but the results of perfusion parameters fit into known values reported in the literature regarding those neoplasms in other intracranial locations [20,23,39].

Abscesses are cystic brain lesions that consist of pus surrounded by a well-vascularized capsule, with low perfusion values within the lesion. On conventional MRI, abscesses may resemble macroadenomas or necrotic tumors, but on PWI imaging these lesions differ significantly, showing low perfusion parameters [38,40,41,42]. This information is crucial, as pituitary abscesses are often identified during the peri-operative or postoperative period [43].

**Figure 9:**
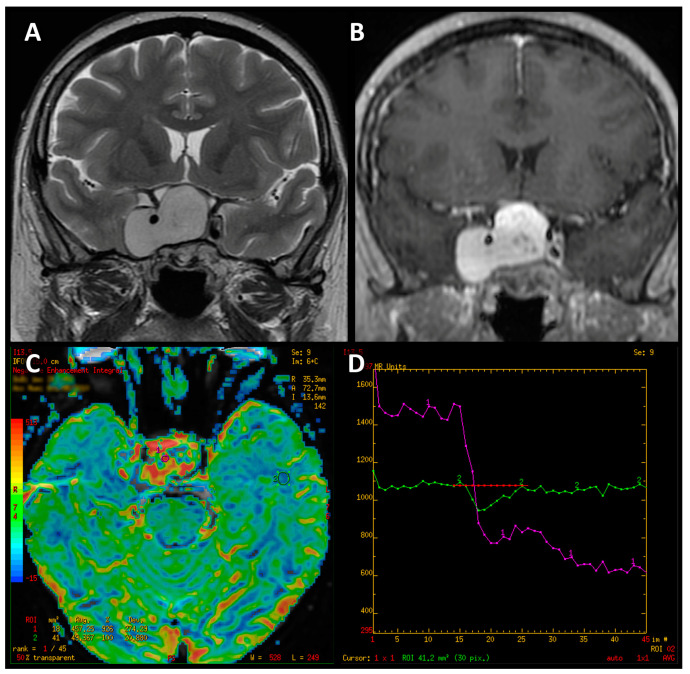
Coronal T2-weighted image of hemangioblastoma (**A**). Contrast-enhanced T1-weighted coronal image (**B**). Color-coded map of cerebral blood volume with the ROI placed in normal-appearing white matter (**C**). T2* signal intensity curve of hemanagioblastoma (purple line), which initially shows an apparent return to the baseline and declines again afterwards (**D**).

Hemangioblastoma revealed high values of perfusion parameters, which is consistent with values reported in the available literature [14,43,44,45,46,47]. However, the impact of hypervascularization of a haemangioblastoma or hemangioma on PWI values requires further research on a larger group—in our study, we only examined single cases of these tumors. We also observed that the perfusion curve obtained for hemangioblastoma presented an unusual shape, atypical for PitNETs as well as meningiomas (Figure 9). It is noteworthy that these PWI-derived features can be useful in the differential diagnosis and enable proper surgical management planning [48,49].

Considering that tumor size may influence the choice of treatment methods, we also investigated the correlation between perfusion parameters and tumor size [49,50]. In our study, we found that larger meningiomas had higher rPH values, which may be caused by high vascularity and the absence of the blood–brain barrier. The lack of other correlations between size and perfusion parameters may be due to the insufficient diversity within the other tumor groups. 

## 5. Conclusions

PWI provides significant additional value to conventional MRI examination of lesions located in the sellar and parasellar regions.

The most appropriable perfusion parameter was rCBV, as it provides substantial information and improves the differential diagnosis. All variants of rCBV parameter values for PitNETs were significantly lower compared to rCBV values of meningiomas. Therefore, this parameter can differentiate the aforementioned groups of tumors with high sensitivity and specificity.

The rPH parameter values of PitNETs were also significantly lower compared to meningiomas, although in our research this parameter presented lower clinical utility than rCBV.

Separate interpretation of rPH has a moderate diagnostic value, but when combined with rCBV value measurement, it supports the differentiation of ambiguous PitNETs and meningiomas on a conventional MR examination. The only perfusion parameter with no statistically significant correlation among all the measured variants was rPSR. The method of calculating the rPSR parameter is not standardized. It varies due to the method of calculation and the variability of the S_1_ point. Thus, further studies are needed to assess the suitability of the rPSR. Nevertheless, the evaluation of the perfusion curve shape can be a useful feature helpful in the differential diagnosis of intracranial tumors including lesion of the sellar and parasellar regions. Finally, according to our experience, PWI should be included in the MR protocol of all intracranial tumors, as it is an accessible and quick imaging technique.

## 6. Limitations 

In our study, we used manual annotations which depend on the user performing the examination. In addition, the tumor must be adequately sized to enable measurement (roughly twice the thickness of slice). The presence of calcifications, and hemorrhagic and cystic components, also precluded accurate measurements. 

## Figures and Tables

**Table 1:** Division into groups by type of tumor.

Type of Tumor	n	%
Non-functional adenomas	51	41.1
Meningiomas	17	13.7
Non-functional adenomas following surgery	12	9.7
Hormone-secreting adenomas	11	8.9
Adamantinomatous type of craniopharyngiomas	9	7.3
Metastasis	5	4.0
Rathke’s cleft cysts	5	4.0
Papillary type of craniopharyngiomas	4	3.2
Lymphomas	2	1.6
Optic chiasm gliomas	2	1.6
Hamartomas	2	1.6
Cavernous hemangioma	1	0.8
Hemangioblastoma	1	0.8
Intrasellar abscess	1	0.8
Teratoma maturum	1	0.8

**Table 2:** Analysis of receiver operating characteristic (ROC) curves for rCBV parameter.

No.	AUC	Parameter	Cut-Off	Compared Tumors
1	0.882	rCBV’2	4.57	Meningiomas vs. Hormone-secreting pituitary adenomas
2	0.872	rCBV’1	3.54	Meningiomas vs. Hormone-secreting pituitary adenomas
3	0.834	rCBV’3	5.29	Meningiomas vs. Hormone-secreting pituitary adenomas
4	0.833	rCBV’1	4.27	Meningiomas vs. Non-functional adenomas followed after surgery
5	0.824	rCBV’2	4.53	Meningiomas vs. Non-functional adenomas followed after surgery
6	0.797	rCBV’1	3.45	Non-functional adenomas vs. Meningiomas
7	0.790	rCBV’2	4.52	Non-functional adenomas vs. Meningiomas
8	0.787	rCBV’3	5.16	Non-functional adenomas vs. Meningiomas
9	0.755	rCBV’3	5.08	Meningiomas vs. Non-functional adenomas following surgery

’1 = mean perfusion parameters for the whole tumor. ’2 = mean of maximum perfusion parameters. ’3 = maximum perfusion values; rCBV parameter—relative cerebral blood volume.

**Table 3:** Analysis of receiver operating characteristic (ROC) curves for rPH parameter.

No.	AUC	Parameter	Cut-Off	Compared Tumors
1.	0.711	rPH’2	1.92	Meningiomas vs. Hormone-secreting pituitary adenomas
2	0.694	rPH’3	2.37	Non-functional adenomas vs. Meningiomas
3	0.679	rPH’3	4.64	Meningiomas vs. Hormone-secreting pituitary adenomas
4	0.676	rPH’2	1.96	Non-functional adenomas vs. Meningiomas

’2 = mean of maximum perfusion parameters. ’3 = maximum perfusion values; rPH parameter—relative peak height.

**Table 4:** Mean values of tumor size.

Type of Tumor	Mean TR	Mean CC	Mean AP
Non-functional adenomas	2.43	2.58	2.21
Meningiomas	2.97	2.70	2.98
Non-functional adenomas following surgery	2.56	2.67	2.34
Hormone-secreting adenomas	3.13	3.31	2.63
Adamantinomatous type of craniopharyngiomas	2.71	3.30	2.72
Metastasis	2.68	2.00	2.42
Rathke’s cleft cysts	1.22	1.42	0.86
Papillary type of craniopharyngiomas	2.65	2.93	2.70
Lymphomas	1.70	1.75	1.60
Optic chiasm gliomas	2.80	2.35	2.85
Hamartomas	2.10	1.60	1.30
Cavernous hemangioma *	2.20	2.90	2.40
Hemangioblastoma *	3.60	2.50	2.40
Intrasellar abscess *	2.00	2.10	1.50
Teratoma maturum *	2.50	3.00	2.20

TR—transverse dimension, CC—cranio-caudal dimension, AP—anterior-posterior dimension, * single tumors for which the mean values were not calculated.

**Table 5:** Measurements of perfusion parameters of tumors group consisting of ≤5 subjects.

Type of Tumor	n	Mean rCBV	Max rCBV	Mean rPH	Max rPH
Metastasis	5	3.64	7.77	1.80	3.65
Rathke’s cleft cyst	5	3.12	4.23	0.31	0.58
Papillary type of craniopharyngioma	4	2.25	5.24	1.94	5.06
Lymphoma	2	1.17	2.15	0.73	1.99
Optic chiasm glioma	2	3.40	6.24	3.33	6.62
Hamartoma	2	1.09	1.59	3.55	3.76
Cavernous hemangioma	1	2.07	5.07	0.22	0.40
Hemangioblastoma	1	4.22	9.21	2.90	7.03
Intrasellar abscess	1	0.63	0.79	0.73	1.26
Teratoma maturum	1	3.50	9.19	0.96	1.90

## Data Availability

Data available on request.
